# Joint cooperative caching and power control for UAV-assisted internet of vehicles

**DOI:** 10.1038/s41598-024-59823-9

**Published:** 2024-04-23

**Authors:** Weiguang Wang, Yang Liu, Yusheng Dai, Yixin He

**Affiliations:** 1 School of Information Engineering, Henan University of Science and Technology, Luoyang, China; 2 School of Electronics and Information, Northwestern Polytechnical University, Xian, 710129 China; 3 China Electronics Standardization Institute, Beijing, China; 4 College of Information Science and Engineering, Jiaxing University, Jiaxing, 314001 China; 5 Jiaxing Key Laboratory of Smart Transportations, Jiaxing, 314001 China

**Keywords:** Engineering, Electrical and electronic engineering

## Abstract

In view of the current problems of spectrum resource scarcity, return congestion, and insufficient energy utilization in the unmanned aerial vehicle (UAV)-assisted Internet of Vehicles (IoV), this paper investigates the cooperative caching and power control, and proposes a joint optimization method to improve the overall Energy Efficiency (EE) . In this method, we first propose a communication establishment threshold to control the V2V communication distance and serve as a joint optimization factor. Then we derive the closed form expressions of offloading ratio and EE of the UAV-assisted IoV, and formulate the optimization problem of maximizing EE. Due to the coupling relationship between caching strategy and transmission power, it is difficult for us to directly solve the optimization problem. Furthermore, we propose an alternating optimization algorithm for solving the optimization problem. Finally, the experimental simulation compare the propose joint optimization method with other existing optimization methods, and the simulation results prove the effectiveness and superiority of the propose joint optimization method.

## Introduction

In recent years, with the increase in the number of motor vehicles and the increase popularity of vehicle applications, the unmanned aerial vehicle (UAV)-assisted Internet of Vehicles (IoV) has received widespread attention in the industry^1–5^. The UAV-assisted IoV can achieve information exchange between Vehicles and Infrastructure (V2I), Vehicle to Vehicle (V2V) and Vehicle to Pedestrian (V2P) through onboard wireless communication devices^6–8^. Mobile edge caching technology can cache content to Cached Vehicles (CV) closer to the RV, which can reduce network transmission latency and power consumption, improving resource utilization^9^. In the UAV-assisted IoV networking, UAV can provide content awareness and coordination services for vehicles, which can increase the efficiency of collaborative caching between vehicles. In addition, when the inter-vehicle caching cannot meet the needs of the Requesting Vehicles (RVs), UAV can directly provide services to RVs. Therefore, applying mobile edge caching technology to the UAV-assisted IoV network can enable real-time information exchange and resource sharing between vehicles, and provide intelligent personalized services according to RVs needs^10^. It can not only minimize the probability of traffic congestion and losses caused by traffic accidents, but also provide RVs with various entertainment information, thereby better realizing the intelligence of transportation systems. Therefore, how to optimize the caching strategy and improve the service time of the UAV-assisted IoV network will be an important scientific problem.

Encouraged by the aforementioned scientific problem, many scholars have conducted extensive research on the cache-enabled UAV-assisted IoV. Specifically, the authors in literature^11^ studied the gains achieved by proactive caching in Roadside Units (RSUs) where we take into consideration the effect of the vehicle velocity on the optimal caching decision. Information about the user demand and mobility is harnessed to cache some files in RSUs, which will communicate with vehicles traversing along the visited roads before the actual demand. The authors’ main objective is to minimize the total network latency. Zhao et al.^12^ proposed an effective caching scheme in V2V communication scenarios based on the similarity and group nature of user activities to improve the content hit ratio. Jiang et al.^13^ proposed a collaborative content allocation and transmission framework to minimize average content download latency. Through interacting with the environment, the vehicle or V2V link can learn the optimal policy based on the strategy gradient and make the decision to select the optimal sub-band and the transmitted power level. Ndikumana et al.^14^ studied an active caching scheme based on deep learning, using a multi-layer perceptron (MLP) method to predict the popularity of content within the coverage area of mobile edge computing servers, in order to determine the content that needs to be downloaded from mobile edge computing servers to vehicles. With the support of long short-term memory neural networks, Ref.^15^ designed an active caching scheme based on Q-learning. The above research improves the performance of the UAV-assisted IoV by optimizing different indicators, but the optimization of a single indicator has very limited effect on improving network performance.

As is well known, there is an interactive relationship between the relevant indicators in the network (such as larger transmission power can increase transmission probability, while it also increases power consumption)^16^. Pure optimization of a single indicator, although it improves network performance to a certain extent, cannot meet the massive data demand in future communication. Therefore, researchers have begun to explore the advantages that comprehensive optimization brings to the UAV-assisted IoV. Yao^17^ aimed to minimize network traffic costs by jointly optimizing content placement and storage allocation in the Internet of Things. The authors formulate the joint problem as an integer linear programming (ILP) model with the objective to minimize the total network traffic cost. The storage allocation problem and content placement problem are constrained by caching storage budgets and cache capacities, respectively. Liang et al.^18^ studied the spectrum and power allocation problem in device-to-device-enabled vehicular networks, where CSI of vehicular links is only reported to the BS periodically. The authors maximize the sum throughput of all V2I links while guaranteeing the reliability of each V2V link with the delayed CSI feedback. Wang et al.^19^ proposed a collaborative caching strategy for content request prediction based on IoV, which pre-caches vehicle request content with a higher probability in other vehicles or roadside units to reduce content acquisition delay and improve cache hit ratio. Zhang et al.^20^ designed an online vehicle caching strategy based on the EE optimization and vehicle mobility analysis, where the interaction between CVs and mobile vehicles are modeled as a two-dimensional Markov process to characterize the network availability of mobile vehicles. Although literature^17–19^ has to some extent improved the performance of vehicle networking, they have not achieved the true goal of joint optimization. So the network performance of the UAV-assisted IoV may not reach its optimal state. Therefore, we need to comprehensively consider the interaction between factors that improve the performance of the UAV-assisted IoV network, in order to solve the issues of scarce network spectrum, small battery capacity, privacy security, limited storage space and low resource utilization. Table 1 summarizes the research on optimization methods mentioned above. Single optimization method is a method for single factor optimization, which is significantly inferior to joint optimization. Staged joint optimization is actually a suboptimal optimization method, which separates independent optimization factors in order. The joint optimization method is to obtain a pair of optimal factor combinations that maximize network performance. However, the joint optimization methods in Refs.^16,20^ are not applicable to cache-enabled UAV-assisted IoV network.

**Table 1:** Related research based on optimization methods.

Optimization methods	References	Disadvantages
Single optimization methods	^11–14^	Limited improvement in network performance
Staged joint optimization methods	^17–19^	Suboptimal optimization methods
Joint optimization methods	^16,20^	Not applicable to edge caching strategy

Based on the above background, this paper proposes a joint optimization method to jointly optimize the caching strategy and transmission power, in order to comprehensively improve the EE and data offloading performance of the UAV-assisted IoV network. The main contributions of this paper are summarized as follows:

1. Based on the theory of random geometry, we have theoretically derived the network EE and the data offloading ratio of UAV-assisted IoV, and obtained their closed form expressions. In addition, We introduced a V2V establishment threshold to strictly set the conditions for the V2V link, and used it as a factor for joint optimization of caching strategy and transmission power.
2. According to the results of theoretical derivation, we have formulated an optimization problem for maximizing the EE of UAV-assisted IoV. We aimed to jointly optimize caching strategies and transmission power to comprehensively improve the EE. Therefore, we proposed a joint optimization algorithm to iteratively solve the formulated optimization problem. The joint optimization algorithm is based on two sub-optimization problems(the cache strategy optimization problem and the transmission power optimization problem), which can ensure that the EE converges to the maximum.
3. The network performance of the proposed joint optimization method was compared with TIOM and TUB optimization methods using the same parameter simulation, and the results proved the superiority and effectiveness of the proposed joint optimization method.

The other sections of this paper are summarized as follows: The “System model of cache-enabled UAV-assisted IoV” is about network models and content access strategies. The “The theoretical analysis and problem formulation” is the theoretical derivation and problem formulation of the UAV-assisted IoV. The “Joint optimization algorithm” proposes the joint optimization algorithm for caching strategy and transmission power. The “Simulation and numerical results” is simulation verification and result analysis. The conclusion of this paper is in the “Conclusion”.

## System model of cache-enabled UAV-assisted IoV

This section mainly introduces the network model and content access model for caching-enabled UAV-assisted IoV.

### The network model

**Figure 1:**
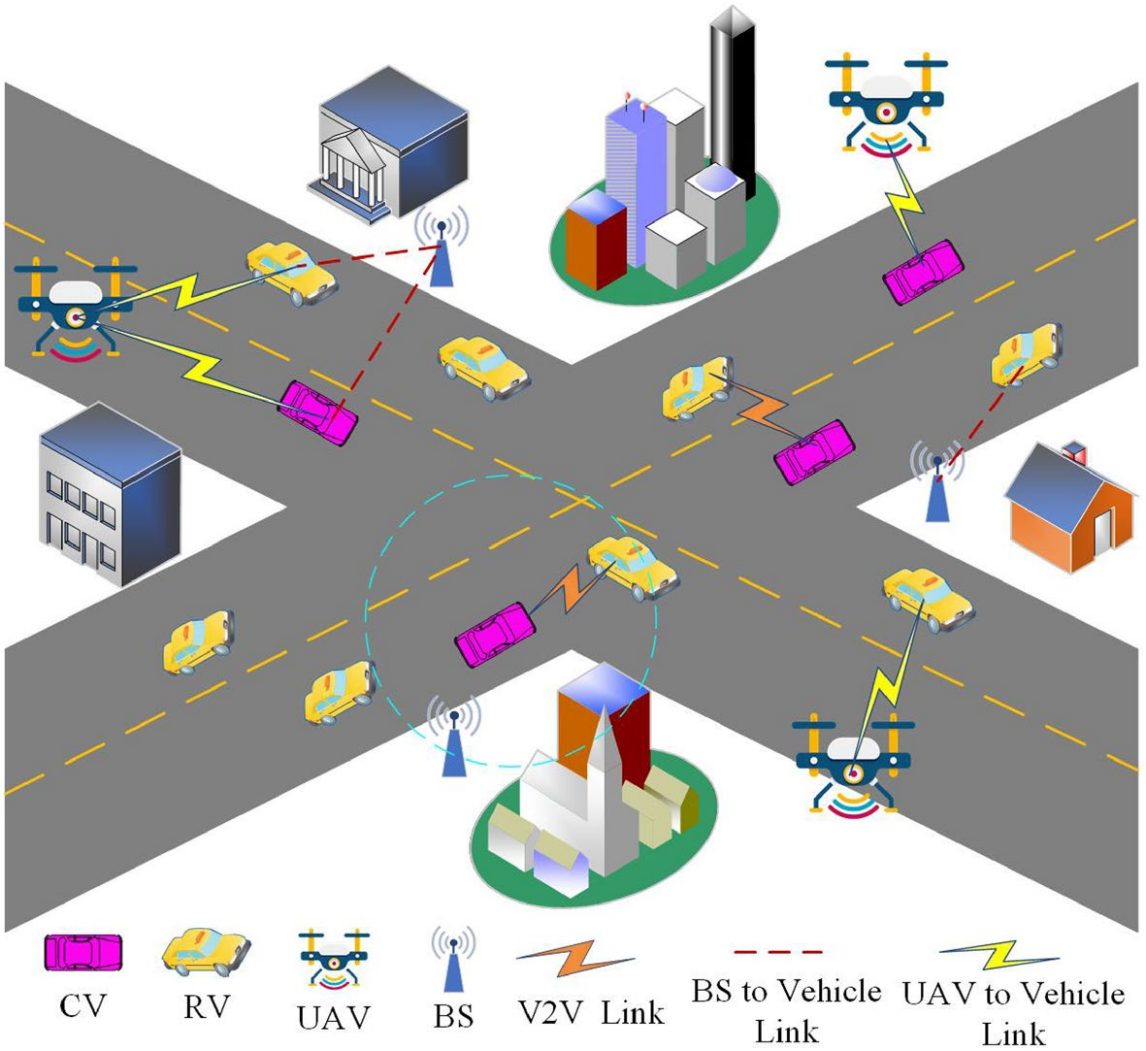
The cache-enabled UAV-assisted IoV.

As shown in Fig. 1, we consider a cache-enabled UAV-assisted IoV consisting of UAVs, CVs and RVs, where both UAV and CVs are capable of storing content in their local caches. When contents are cached at CVs, V2V communications can be utilized for content sharing and delivery among RVs. The cache-enabled UAV-assisted IoV model simulates a busy urban area or a multi-road intersection area, where the vehicle position obeys a homogeneous poisson point processes (HPPP)^21^. The density of CVs and RVs can be represented as $\lambda _p$ and $\lambda _r$, respectively. The vehicles can use device-to-device (D2D) communication technology for local V2V communication. Each CV adopts the same transmission power $P_t$. The Base Station (BS) or UAV can detect the status information of CVs and RVs to assist V2V communication^22^. Due to environmental factors, the signal transmission between the transmitter and receiver will be affected by varying degrees of fading. For large-scale fading, it can be calculated as $r^{-\alpha }$, where *r* represents the communication distance between vehicles and $\alpha$ represents the path loss factor $(\alpha \ge 2)$. Due to our consideration of high-rise urban scenes composed of many ground obstacles, this paper adopts Rayleigh fading for small-scale fading, which follows a unit mean exponential distribution, i.e. $G \sim \exp (1)$. This assumption has been widely used for vehicular communications^23,24^.

Let $\mathscr {F}=[1,2, \ldots , F]$ represent the content set, where 1-st represents the most popular content and *F* represents the least popular content. The number of future content requests is predicted based on the previous frequency of downloads and user interests. Many related studies have shown that the probability of content requests follows a Zipf distribution^19,25–20^. In zipf distribution, the contents are ranked according to the predicted number of requests and the popularity of a content is calculated as a ratio where the numerator represents the rank of the specific content and the denominator represents all content objects. Therefore, the request probability for content can be calculated as $p_f=f^{-\varepsilon } / \sum _{i=1}^F i^{-\varepsilon }$, where $\varepsilon$ represents the content popularity factor. The larger the value of $\varepsilon$, the more focused the content requested. The $\varepsilon =0$ means that the content has the same request probability. Due to the randomness of content and vehicle location, we consider the set $\textbf{q}=\{q_1, \ldots , q_f, \ldots , q_F\}$ as the caching strategy for CVs. This will be the focus of our work to increase the data offloading ratio of UAV-assisted IoV. Each cached vehicle will have its own cache content according to the designated cache strategy *q*. According to the HPPP, we model the CVs’ location distribution as $q_f \lambda _p$, which has cached‘ content *f*.

### The content access model

This paper mainly considers two content access modes, namely self-caching and inter-vehicle caching. When RVs fail to obtain content through self-caching and inter-vehicle caching, there will be BS or UAV providing content support for RVs.

1. ${\textbf {Self-caching}}$: when the required content is cached in a self-caching library, RVs will automatically download the required content through self-caching. This is a special caching method that does not cause energy consumption, but is often overlooked.
2. ${\textbf {Inter-vehicle caching}}$: this is a type of cooperative caching, which is also the main research content of this paper. In mode inter-vehicle caching, vehicles can share content through V2V communication, thereby increasing content cache diversity and data offloading ratio, and reducing redundant transmission.

## The theoretical analysis and problem formulation

This section mainly proposes the threshold for establishing V2V communication, and investigates the data offloading ratio and EE of UAV-assisted IoV.

### Data offloading ratio and EE analysis

Since the CV location of cached the content follow a HPPP with the density $q_f \lambda _p$. Therefore, the probability density function of CV transmitting content *f* to RV with the associated distance *r* can be expressed as $f=2 \pi q_f \lambda _p r e^{-\pi q_f \lambda _p r^2}$^27,28^. Due to the random distribution of vehicles in the UAV-assisted IoV, their associated distances may very far. At this point, if increasing the transmission power to establish V2V communication without considering the distance factor, it will increase interference and transmission power consumption, reduce the proximity gain of V2V communication. If the transmission power is not increased, it may lead to transmission failure. Encouraged by the above investigation, we propose a V2V communication establishment threshold $\theta$ $\left( P_t r^{-\alpha }>\theta \right)$ to control the associated distance, in order to balance the contradictory relationship between network data offloading ratio and transmission power consumption. The V2V communication can only be established when the average received power of the RV is greater than $\theta$.

1. The self-caching offloading ratio
  The self-caching mode retrieves content from the self-caching library, so the data offloading ratio of self-caching $Q^{\text {self }}$ is the cache hit ratio, which can be calculated as
  1 $$\begin{aligned}&Q^{\text {self }}=\sum _{f=1}^F p_f q_f. \end{aligned}$$
2. The Inter-vehicle caching offloading ratio
  The Inter-vehicle caching offloading ratio $D_{V 2 V}$ mainly consists of the cache hit ratio $Q_f^{V 2 V}$ and the successful transmission probability $P_{V 2 V}^S$, which can be expressed as
  2 $$\begin{aligned} D_{V 2 V}=\sum _{f=1}^F p_f P_{V 2 V}^S Q_f^{V 2 V}. \end{aligned}$$

The hit ratio of inter-vehicle caching is defined as the probability that the content *f* is cached between vehicles and successfully perceived within the communication distance *r*, which can be calculated as

3 $$\begin{aligned} Q_f^{V 2 V}=1-e^{-\pi \lambda _p q_f r^2}. \end{aligned}$$

Therefore, the probability of all content in the content set $\mathscr {F}=[1,2, \ldots , F]$ being successfully found through the inter-vehicle caching can be calculated as

4 $$\begin{aligned} Q^{V 2 V}=\sum _{f=1}^F p_f\left( 1-e^{-\pi \lambda _p q_f r^2}\right) . \end{aligned}$$

According to Shannon’s theorem, when the RV’s signal-to-interference-and-noise ratio (SINR) $\gamma _f=\frac{P_t g_{0 f} r^{-\alpha }}{I_1+N_0 w}$ exceeds the SINR threshold $\gamma _0$, the RV can correctly recover the transmitted content. Here $g_{0, f}$ represents the channel gain between CV and RV, $I_1=\sum _{i \in \Phi _1 \backslash \subset p_0} P_t g_i r_i^{-\alpha }$ represents interference caused by active CVs in the surrounding area, $N_0 w$ is the noise power received at RV. However, the successful transmission of cached content between vehicles must meet both the SINR threshold and the V2V communication establishment threshold conditions. Therefore, the successful transmission probability of Inter-vehicle caching $P_{V 2 V}^S$ can be expressed as

5 $$ \begin{aligned}   P_{{V2V}}^{S}  &  = {\mathbb{P}}\left[ {P_{t} r^{{ - \alpha }}  \ge \theta ,\gamma _{f}  \ge \gamma _{0} } \right] = {\mathbb{P}\ominus }0 < r \le z,\gamma _{f}  \ge \gamma _{0} ] \\     &  = \int_{0}^{z} {\mathbb{P}} \left( {\gamma _{f}  \ge \gamma _{0} } \right)f(r){\text{d}}r \\     & \mathop  \approx \limits^{{(1)}} \int_{0}^{z} {\mathbb{P}} \left( {g_{{0f}}  \ge P_{t}^{{ - 1}} r^{{ - \alpha }} I_{1} \gamma _{1} } \right)f(r){\text{d}}r \\     & \mathop  = \limits^{{(2)}} \int_{0}^{z} {{\mathbb{E}}_{{I_{1} }} } \left( {{\text{e}}^{{ - P_{t}^{{ - 1}} r^{\alpha } \alpha _{1} \gamma _{1} }} } \right)f(r){\text{d}}r\mathop  = \limits^{{(3)}} \int_{0}^{z} {{\mathscr{L}}_{{I_{1} }} } \left( {P_{t}^{{ - 1}} r^{\alpha } \gamma _{0} } \right)f(r){\text{d}}r, \\  \end{aligned}  $$

where step (1) is a consideration for surrounding interference, step (2) is a consideration for small-scale fading $g_{0 f} \sim \exp (1)$ and step (3) represents the Laplace transform of $I_1$.

Assuming variable $s=P_t^{-1} r^\alpha \gamma _0$ then the Laplace transform of variable $I_1$ can be calculated as

6 $$ \begin{aligned}   {\mathscr{L}}_{{I_{1} }} (s) &  = {\mathbb{E}}_{{\Phi _{1} ,g_{i} }} \left[ {e^{{ - s\sum _{{i \in \Phi _{1} \backslash cp_{0} }} P_{t} g_{i} r_{i}^{{ - \alpha }} }} } \right] \\     &  = {\mathbb{E}}_{{\Phi _{1} }} \left[ {\prod _{{i \in \Phi _{1} \backslash CP_{0} }} \left( {1 + sP_{t} r_{i}^{{ - \alpha }} } \right)^{{ - 1}} } \right] \\     &  = \exp \left\{ { - 2\pi \lambda _{r} Q^{{V2V}} \int_{0}^{\infty } {\left( {1 - \frac{1}{{1 + sP_{t} x^{{ - \alpha }} }}} \right)} x{\text{dx}}} \right\} \\     &  = \exp \left\{ { - 2\pi \lambda _{r} Q^{{V2V}} \int_{0}^{\infty } {\left( {\frac{{y^{{\frac{2}{\alpha } - 1}} }}{{1 + \left( {sP_{t} } \right)^{{ - 1}} y}}} \right)} \frac{1}{\alpha }{\text{d}}x} \right\} \\     &  = \exp \left\{ { - 2\pi ^{2} \lambda _{r} Q^{{V2V}} \left( {sP_{t} } \right)^{{2/\alpha }} \csc \left( {2\pi \alpha ^{{ - 1}} } \right)\alpha ^{{ - 1}} } \right\}. \\  \end{aligned}  $$

Furthermore, the successful transmission probability of Inter-vehicle caching $P_{V 2 V}^S$ can be calculated as

7 $$\begin{aligned} \begin{aligned} P_{V 2 V}^S&=\mathbb {E}\left[ {\text {Pr}}\left( P_t r^{-\alpha } \ge \theta , \gamma _f \ge \gamma _0\right) \right] \\&=2 \pi \lambda _p q_f \int _0^z e^{-\varphi _f r^2} r \text {d} r \\&=\frac{\pi q_f \lambda _p}{Q_f^{V 2 V}}\left( \frac{1-e^{-\varphi _f z^2}}{\varphi _f}\right) , \end{aligned} \end{aligned}$$

where the variable $\varphi _f$ represents $\varphi _f=\pi q_f \lambda _p+2 \pi ^2 \lambda _r \gamma _0^{2 / \alpha } Q^{V 2 V} \csc \left( 2 \pi \alpha ^{-1}\right) \alpha ^{-1}$.

The content offloading ratio is defined as the probability that the content is successfully perceived and transmitted to the RV. Through Eqs. (1) and (2), we can calculate the data offloading ratio of UAV-assisted IoV $P_h$ as

8 $$\begin{aligned} \begin{aligned} P_h&=Q^{\text {self }}+\sum _{f=1}^F p_f P_{V 2 V}^S Q_f^{V 2 V} \\&=\sum _{f=1}^F p_f\left[ q_f+\pi q_f \lambda _p\left( \frac{1-e^{-\varphi _f z^2}}{\varphi _f}\right) \right] . \end{aligned} \end{aligned}$$

We define the EE as the ratio of the number of successfully transmitted content bits per unit time to the total required power consumption. Therefore, the EE can be expressed mathematically as $E E_{\text {total }}=\Psi / E C_{\text {total }}$, where $\Psi =R_t P_h \lambda _p$ represents the number of successfully transmitted content bits per unit time, $E C_{\text {total }}$ represents the total power consumption. Here, we adopt a linear power consumption model, so the total power consumption can be expressed as $E C_{\text {total }}=\lambda _p\left( \frac{1}{\mu } P_t+P_c\right)$. Furthermore, we can calculate the EE of UAV-assisted IoV as

9 $$ \begin{aligned}   EE_{{{\text{total }}}}  &  = \sum\limits_{{f = 1}}^{F} {p_{f} } R_{t} \pi q_{f} \lambda _{p} \left( {\frac{{1 - e^{{ - \varphi _{{1,f}} r^{2} }} }}{{\varphi _{{1,f}} }}} \right)\left( {\frac{\mu }{{P_{t}  + \mu P_{c} }}} \right) \\     &  = \sum\limits_{{f = 1}}^{F} \pi  \lambda _{p} p_{f} R_{t} q_{f} \mu \frac{{1 - e^{{ - \varphi _{f} r^{2} }} }}{{\varphi _{f} \left( {P_{t}  + \mu P_{c} } \right)}} \\  \end{aligned}  $$

### The optimization problem formulation

Based on considerations of the network caching strategy and transmission power, we can describe the optimization problem of maximizing EE as

10 $$\begin{aligned} \begin{aligned} {{\textbf {P0:}} }&\max _{P_t} E E_{\text {total }}=\sum _{f=1}^F \pi \lambda _p p_f R_t q_f \mu \frac{1-e^{-\varphi _f r^2}}{\varphi _f\left( P_t+\mu P_c\right) } \\ {}&\text { subject to } 0 \le P_t \le P_t^{\max },\\ {}&\quad \sum _{f=1}^F q_f \le C, 0 \le q_f \le 1, \forall f . \end{aligned}. \end{aligned}$$

Due to the introduction of exponential terms in Eq. (8), the optimization problem Eq. (10) becomes more complex. Therefore, we propose a joint optimization method to optimize cache policy and transmission power, which is based on two sub-problems: cache strategy optimization and transmission power optimization.

1. The cache strategy optimization problem, which is to optimize and solve the cache strategy under a given transmission power. Based on Eq. (8), we can describe the cache strategy optimization problem as
  11 $$ \begin{aligned}    & {\mathbf{P1}}:\max _{{\mathbf{q}}} P_{h}  \\     & {\text{subject to }}\sum\limits_{{f = 1}}^{F} {q_{f} }  \le C,0 \le q_{f}  \le 1,\forall f. \\  \end{aligned}  $$
  *Proposition 1*: The objective function $P_h$ is a concave function on interval $0 \le q_f \le 1$, and the cache strategy optimization problem **P1** is a convex programming problem.
  **Proof: see Appendix A**
  Therefore, any convex optimization solution is suitable for solving cache strategy optimization problem **P1**. This paper adopts the CVX convex optimization solver for optimal cache strategy **q**, which can quickly converge the original optimization problem to the optimal solution.
2. The transmission power optimization problem, which is to optimize and solve the transmission power under a given cache strategy. Therefore, the optimization problem of the EE regarding transmission power can be modeled as
  12 $$\begin{aligned} \begin{aligned} {{\textbf {P2:}} }&\max _{P_t} E E_{\text {total }}=\sum _{f=1}^F \pi \lambda _p p_f R_t q_f \mu \frac{1-e^{-\varphi _f r^2}}{\varphi _f\left( P_t+\mu P_c\right) } \\&\text { subject to } 0 \le P_t \le P_t^{\max } . \end{aligned}. \end{aligned}$$
  For ease of calculation, we simplify the variables in Eq. (10) by assuming $\xi \left( P_t\right) =\varphi _f P_t / \theta$ and $v=\varphi _f \mu P_c$. Therefore, Eq. (10) can be rewritten as
  13 $$\begin{aligned} \begin{aligned} E E_{\text {total }}=\sum _{f=1}^F \pi \lambda _p p_f R_t q_f \mu \frac{\left( 1-e^{-\xi \left( P_t\right) }\right) }{\xi \left( P_t\right) \theta +v}. \end{aligned} \end{aligned}$$
  Furthermore, take the first-order derivative of function $\Gamma _f\left( P_t\right) =\frac{1-e^{-\xi \left( P_t\right) }}{\xi \left( P_t\right) \theta +v}$ with respect to $P_t \ge 0$, which can be expressed as
  14 $$ \begin{aligned}   \frac{{{\text{d}}\Gamma _{f} \left( {P_{t} } \right)}}{{{\text{d}}P_{t} }} &  = \frac{{{\text{d}}\Gamma _{f} \left( {P_{t} } \right)}}{{{\text{d}}\xi \left( {P_{t} } \right)}}\frac{{{\text{d}}\xi \left( {P_{t} } \right)}}{{{\text{d}}P_{t} }} \\     &  = \frac{{\varphi _{f} }}{\theta }\frac{{\phi _{f} \left[ {\xi \left( {P_{t} } \right)} \right]}}{{\left( {\xi \left( {P_{t} } \right)\theta  + v} \right)^{2} }} \\  \end{aligned}  $$
  where the function $\phi _f\left[ \xi \left( P_t\right) \right]$ represents $\phi _f\left[ \xi \left( P_t\right) \right] =\xi \left( P_t\right) \theta e^{-\xi \left( P_t\right) }+(v+\theta ) e^{-\xi \left( P_t\right) }-\theta$.
  We can easily calculate the first-order derivative of function $\phi _f\left[ \xi \left( P_t\right) \right]$ with respect to $P_t \ge 0$ as
  15 $$\begin{aligned} \phi _f^{\prime }\left[ \xi \left( P_t\right) \right] =-\xi \left( P_t\right) \theta e^{-\xi \left( P_t\right) }-v e^{-\xi \left( P_t\right) } \le 0. \end{aligned}$$
  By analyzing formula Eq. (15), we can conclude that function $\phi _f\left[ \xi \left( P_t\right) \right]$ is a monotonically decreasing function. Furthermore, since $\left( \xi \left( P_t\right) \theta +v\right) ^2$ is an increasing function, we can conclude that $\textrm{d} \Gamma _f\left( P_t\right) / \textrm{d} P_t$ is a monotonically decreasing function. We can easily determine that variable $\xi \left( P_t\right) \rightarrow 0$ yields function $\left. \phi _f\left[ \xi \left( P_t\right) \right] \right| _{\xi \left( P_t\right) \rightarrow 0}=v \ge 0$, and variable variable $\quad \xi \left( P_t\right) \rightarrow \infty$ yields function $\left. \phi _f\left[ \xi \left( P_t\right) \right] \right| _{\xi \left( P_t\right) \rightarrow \infty }=-\theta \le 0$. Therefore, the function $\Gamma _f\left( P_t\right)$ monotonically increases and then decreases with respect to $0<P_t \le P_{\max }$. The optimal transmission power $P_t^*$ can be obtained by solving Eq. (15).
  16 $$ \begin{aligned}    & \frac{{{\text{d}}EE_{{{\text{total }}}} }}{{{\text{d}}P_{t} }} = 0 \\     & \sum\limits_{{f = 1}}^{F} \pi  \lambda _{p} p_{f} R_{t} q_{f} \mu \frac{{\varphi _{f} }}{\theta }\frac{{\phi _{f} \left[ {\xi \left( {P_{t} } \right)} \right]}}{{\left( {\xi \left( {P_{t} } \right)\theta  + v} \right)^{2} }} = 0 \\  \end{aligned}  $$
  By analyzing Eq. (16), we can solve for the optimal transmission power through mathematical expression $\phi _f\left[ \xi \left( P_t\right) \right] =0$. The calculation result can be expressed as
  17 $$ \begin{aligned}    & \xi \left( {P_{t} } \right)\theta e^{{ - \xi \left( {P_{t} } \right)}}  + (v + \theta )e^{{ - \xi \left( {P_{t} } \right)}}  - \theta  = 0 \\     & \frac{{v + \theta }}{\theta } = e^{{\xi \left( {P_{t} } \right)}}  - \xi \left( {P_{t} } \right) \\  \end{aligned}  $$
  Based on the above analysis, it can be concluded that the function $\phi _f\left[ \xi \left( P_t\right) \right]$ is a monotonically decreasing function with respect to $0<P_t \le P_{\max }$. So we can easily solve for the optimal transmission power $P_t^*$ using the bi-section method.

## Joint optimization algorithm

The increase of transmission power can improve the successful transmission probability and the communication range, while also leading to the increase of inter-vehicles interference and power consumption. The increase in communication range can improve the probability of content being successfully offloaded in the UAV-assisted IoV. Taking inspiration from this, we propose a V2V communication establishment threshold $\theta$ to control communication distance, while serving as a factor for joint optimization of caching strategy and transmission power. In other words, we are attempting to achieve the optimal balance between caching strategy and transmission power through joint optimization methods.

**Figure:**
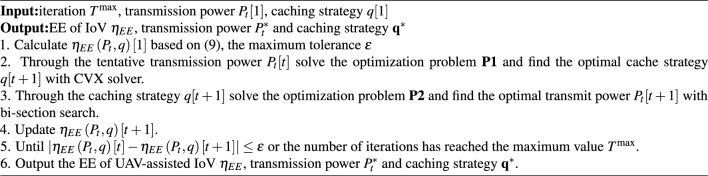
**Algorithm 1** Joint optimization algorithm

**Figure 2:**
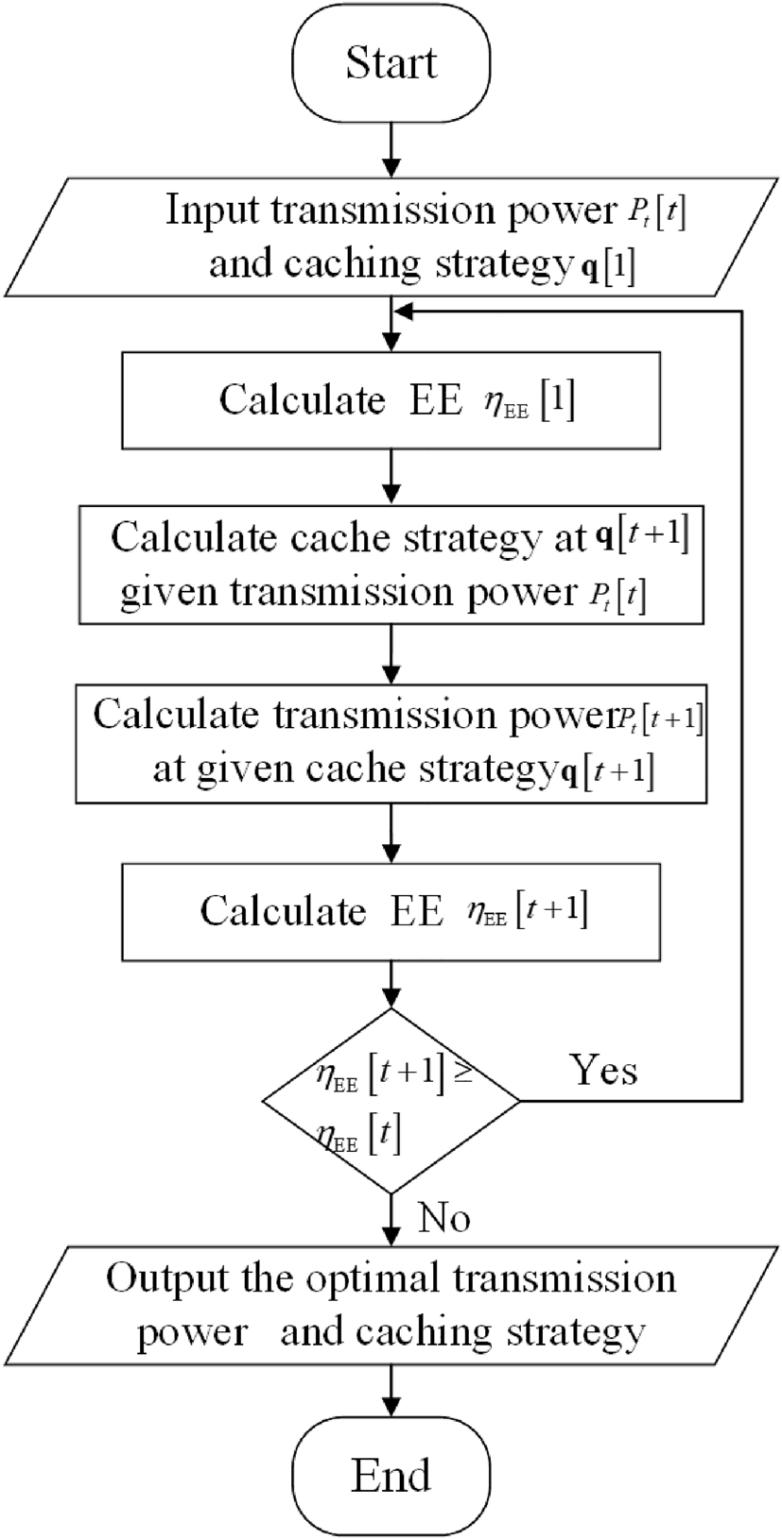
The flowchart of the algorithm.

Based on the theoretical analysis in the previous section, we propose a joint optimization algorithm to iteratively solve optimization problems **P1** and **P2**. The detailed solution process is shown in Algorithm 1. At the beginning of the joint optimization algorithm, we attempted to use transmission power ${P_t}$ to calculate the caching strategy $\mathbf{{q}}$. For the tentative transmission power${P_t}$, we have found a optimal caching strategy $[\mathbf{{q}}^*]$that is better than the caching strategy $\mathbf{{q}}$. Then we found a transmission power ${P_t}^*$ for the tentative optimal caching strategy $[\mathbf{{q}}^*]$. Repeat the alternating optimization of the above two steps until the EE is maximized. The line 1 is the initial calculation of the cache strategy, the transmission power and the EE. The line 2 is to solve optimization problem **P1** under a given transmission power. In other words, for the tentative transmission power $P_t[t]$, find an optimal caching strategy $q[t+1]$. The line 3 is to solve optimization problem **P2** under a given caching strategy, which can obtain the optimal transmission power. Repeat calculation line 2-5 until the EE reaches the maximum value, and output a set of optimal caching strategies and transmission power. In order to facilitate understanding of the algorithm structure, we have provided a flowchart for Algorithm 1. The algorithm flowchart is shown in Fig. 2.

Computational complexity: the complexity analysis of **Algorithm 1** is mainly derived from two parts: cache strategy optimization and transmission power optimization. The complexity of cache strategy optimization can be calculated as $[O\left( {F} \right) ]$, where *F* is the number of contents to be calculated for each iteration. The optimal transmission power is obtained through bi-section search and converges to a solution with a fault tolerance of $\sigma$. Therefore, the complexity of optimizing transmission power is calculated as $[O\left( {{{\log }_2}\left( {{{{P_{\max }}} / {{P_t}}}} \right) } \right)$, where ${P_{\max }}$ represents the maximum transmission power of the CV. Therefore, the total computational complexity of **Algorithm 1** can be approximated as $[\left( {O\left( {F } \right) + O\left( {{{\log }_2}\left( {{{{P_{\max }}} / {{P_t}}}} \right) } \right) } \right) \cdot {T^{\max }}$, where ${T^{\max }}$ is the maximum number of iterations.

## Simulation and numerical results

This section verifies the effectiveness of the proposed joint optimization method through simulation experiments. The simulation experiment considers a busy urban area or a multi-road intersection area, where the vehicle position obeys a HPPP. In the simulation experiment, we mainly explored the effects of factors such as content popularity, association distance and vehicle density on the EE. The parameter settings for the simulation experiment are shown in Table 2. In addition, we validated the superiority of the proposed joint optimization method (with legend “TPJOM”) compared to the independent optimization method and the uniform-baseline. The independent optimization method is a phased joint optimization method (with legend “TIOM”)^29^, in which each factor is independently optimized. By comparing the proposed joint optimization method with TIOM, we aim to verify the superiority of EE. The uniform-baseline is a caching method that caches all contents with equal probability (with legend “TUB”)^30^, which serves as the benchmark for simulation experiments.

**Table 2:** Simulation parameters.

Parameters	Value
Intensity of CVs, $\lambda _p$	0.6 $\textrm{m}^{-2}$
Intensity of RVs, $\lambda _r$	0.4 $\textrm{m}^{-2}$
Transmission power of CV, $P_t$	0.25 W
2V bandwidth, *W*	20 MHz
Path loss exponent, $\alpha$	2
Noise power, $\sigma ^2$	$- 95$ dBm/Hz
Number of contents, *F*	10 files
Each user cache capacity	1 file
Circuit power consumption, $P_c$	115.9 mW
Power amplifier efficiency, *v*	0.1
Zipf parameter, $\varepsilon$	0.6, 1

**Figure 3:**
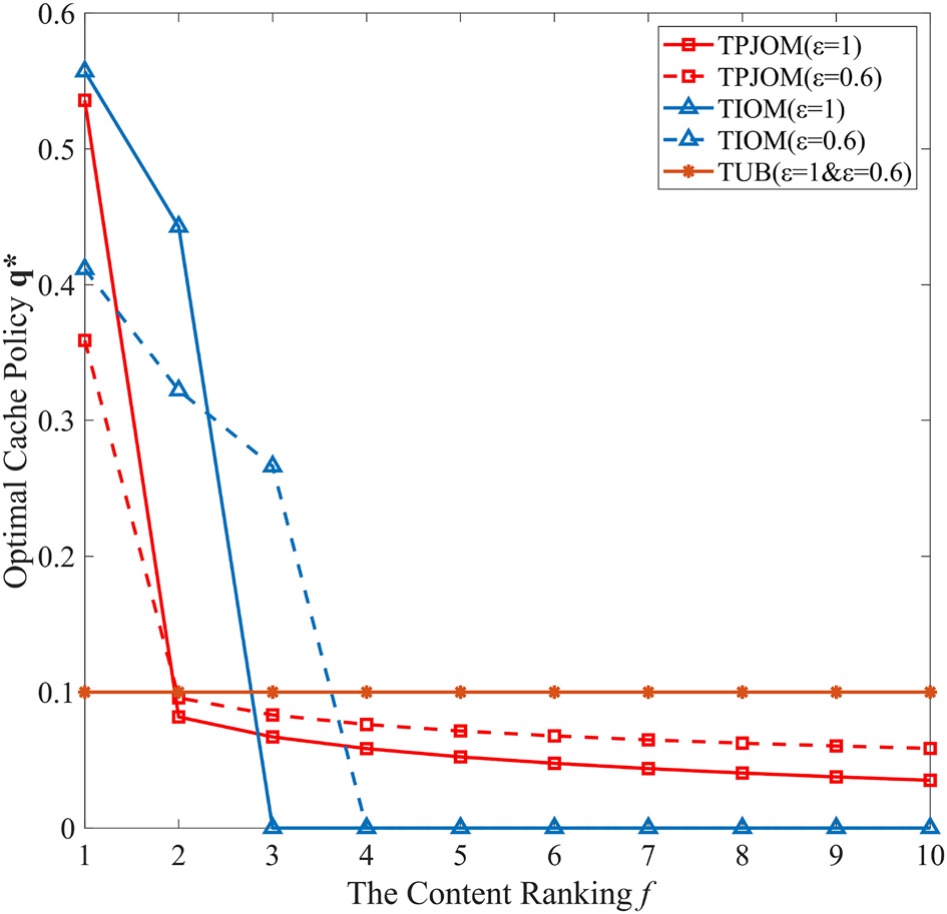
The content caching probability with different Zipf parameters.

In Fig. 3, we describe the content distribution of different caching strategies as the Zipf factor changes. Similarly, both TPJOM and TIOM increase the caching probability for content with higher popularity rankings. As the Zipf factor $\varepsilon$ increased, TPJOM and TIOM also increase the probability of high ranking content. However, TIOM only cached the top three ranked contents. TPJOM has a high probability of caching the top ranked content and a low probability of caching less popular content. Although caching high ranked content can increase the network data offloading ratio, which is consistent with the actual network request situation. But from the perspective of the network EE, the TPJOM not only considers the network data offloading ratio, but also considers the diversity of cached content. The TUB cached all content with equal probability.

**Figure 4:**
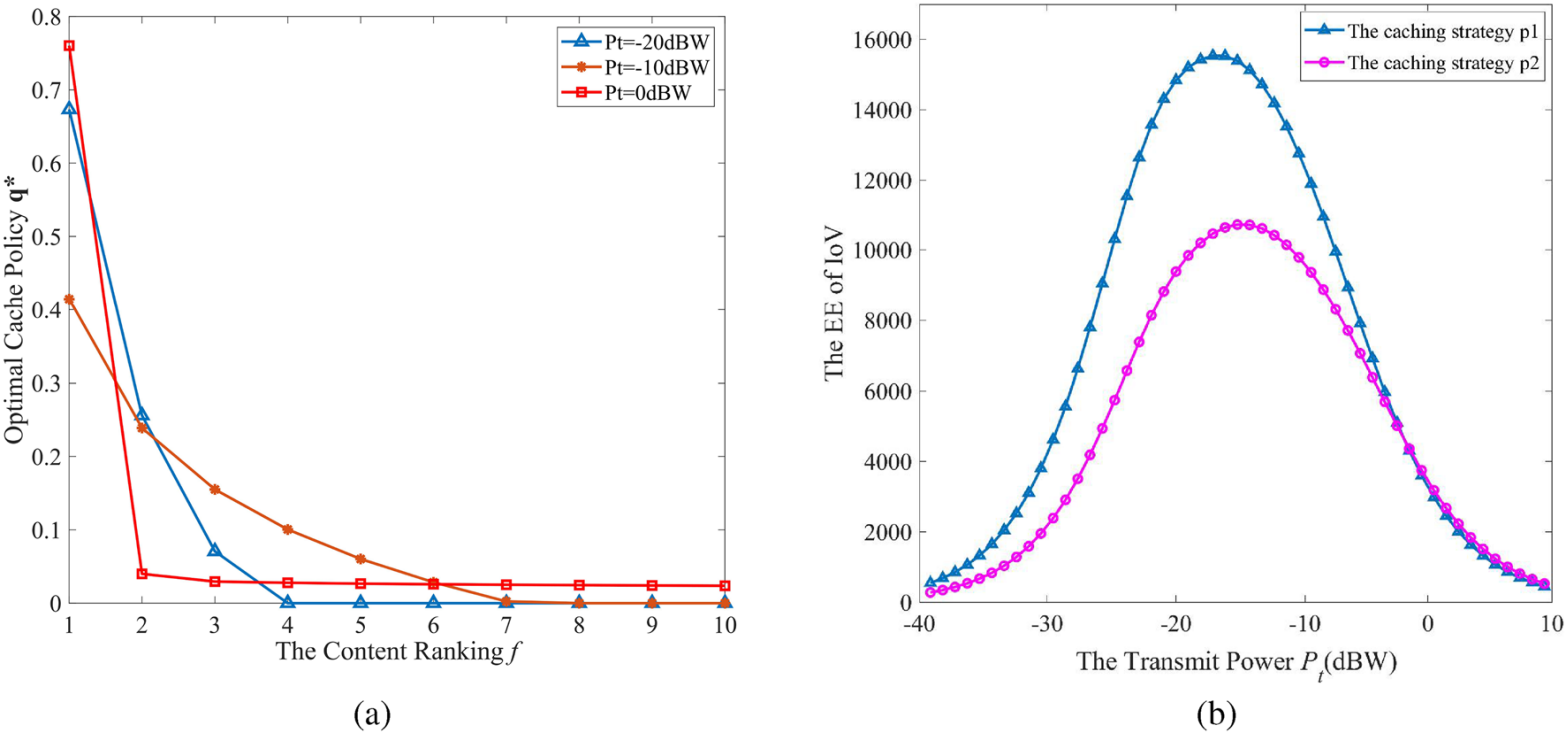
The coupling relationship between caching strategy and transmission power.

In Fig. 4, we analyzed the interaction between caching strategy and transmission power on the EE of UAV-assisted IoV. From Fig. 4a, we can see that as the transmission power changes, the caching strategy also changes. The higher transmission power of CV, the more content is cached. In Fig. 4b, the EE of UAV-assisted IoV with different caching strategies increases with the increase of transmission power, first reaching the maximum value and then rapidly decreasing. Therefore, there exists an optimal transmission power to maximize the EE of UAV-assisted IoV. The simulation results verify the interaction relationship between cache strategy and transmission power in theoretical analysis. Meanwhile, it also indicates the effectiveness of V2V communication establishment threshold $\theta$. Furthermore, we can use $\theta$ to control the V2V communication distance and jointly optimize the caching strategy and transmission power.

**Figure 5:**
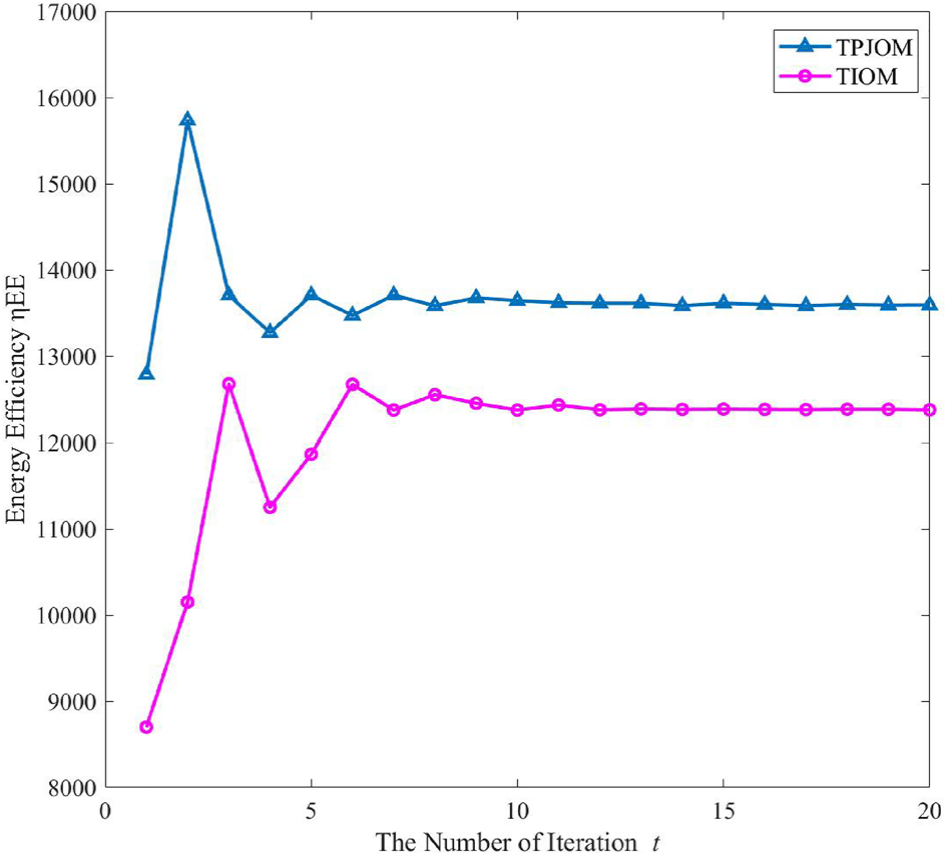
The convergence analysis of algorithms.

In Fig. 5, we analyzed the convergence of TPJOM and TIOM when transmitting power ${P_t} = - 16\text{{ dBW}}$ and popularity parameter $\varepsilon = 0.6$. The horizontal axis in the figure shows that as the number of iterations *t* increases, the EE of TPJOM and TIOM can quickly approach a fixed value. TPJOM can reach the maximum EE by iterating 10 times, while TIOM needs to iterate more than 10 times to stabilize the EE. From the vertical axis, we can see that the EE curve of TPJOM is significantly better than that of TIOM. Because TPJOM jointly considered the impact of V2V establishment threshold and SINR threshold on EE, while TIOM only considered the impact of SINR threshold.

**Figure 6:**
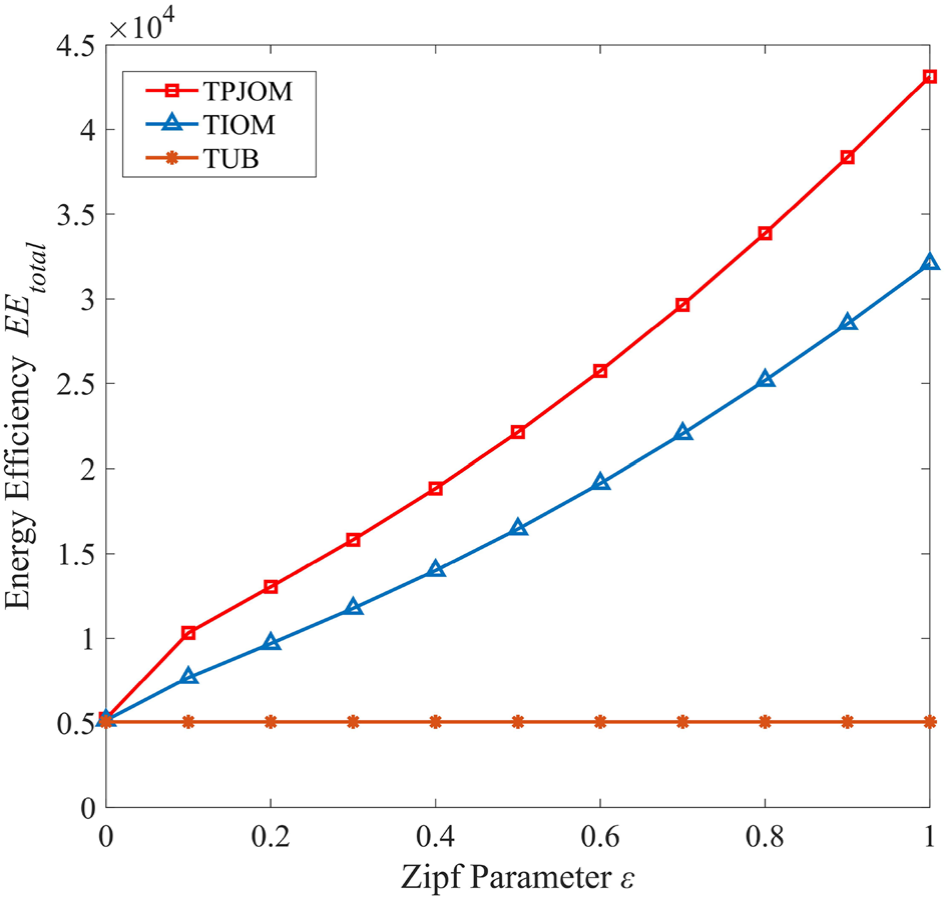
The EE of different caching strategies versus Zipf parameters.

In Fig. 6, we investigated the EE of TPJOM, TIOM and TUB varies with the Zipf parameters. From the simulation results in Fig. 6, we can see that the EE of TPJOM and TIOM increases rapidly with the increase of Zipf parameters. The TUB caches all content with an equal probability, so its EE does not change with changes in content popularity. This also indicates that pure caching strategies do not significantly improve network performance, we must design effective caching strategies to significantly improve network performance. In addition, the EE curve of TPJOM is better than TIOM. This is because our designed caching strategy takes into account cache diversity and adjusts transmission power as the caching strategy changes. Similarly, adjust the content caching strategy based on the distance of CV collaboration. Therefore, the improvement of EE in UAV-assisted IoV requires comprehensive consider the impact situation and jointly optimize multiple factors.

**Figure 7:**
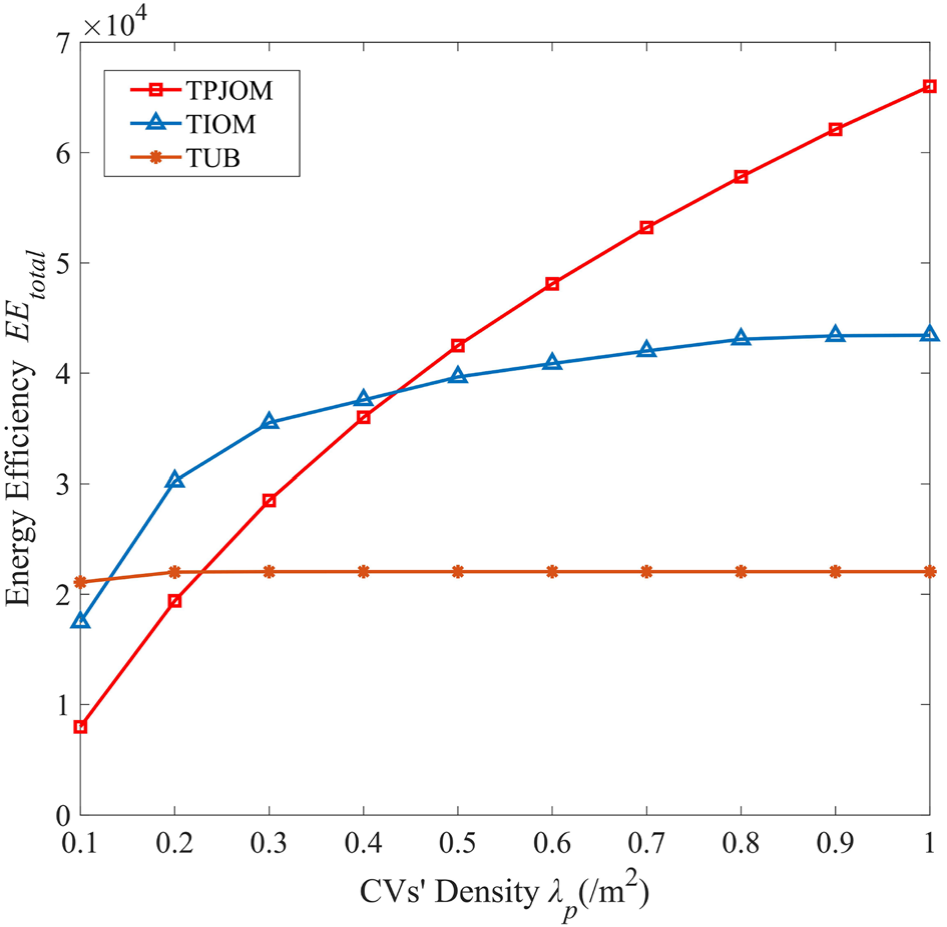
The EE of different caching strategies versus CV’s density.

Figure 7 investigates the variation of EE with CV’s density for different caching strategies. From the graph, we can see that the EE of TPJOM and TIOM increases with the increase of CV’s density. Due to TUB caching all content with an equal probability, the EE of TUB does not change significantly. At the beginning of the curve, the CV’s density is relatively low. Due to the limitation of association distance by TPJOM, the EE of TPJOM is lower than that of TIOM and TUB. When the CV’s density reaches a certain level, the EE of TPJOM will be significantly higher than TIOM and TUB. Due to the increase in CV’s density, the probability of RV finding the required content in the surrounding area has increased. Due to TIOM only caching the top ranked content, its EE gradually tends to balance with the increase of CV’s density. This also indicates that the improvement of EE in the UAV-assisted IoV not only needs to consider content popularity, but also the diversity of cached content.

**Figure 8:**
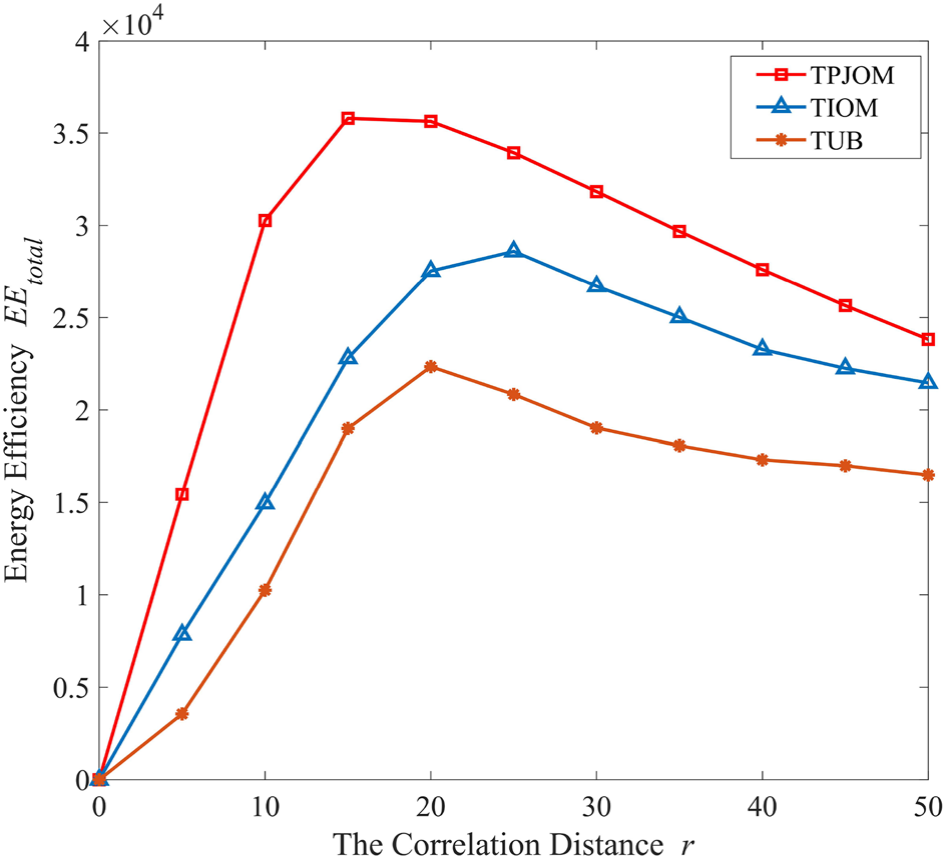
The EE of different caching strategies versus correlation distance.

In Fig. 8, we investigated the EE of TPJOM, TIOM and TUB varies with the correlation distance. From the simulation results in Fig. 8, we can see that the EE of all caching strategies will sharply increase first and then decrease with the increase of correlation distance. The EE curve of TPJOM is significantly better than that of TIOM and TUB. This is because the proposed V2V communication establishment threshold $\theta$ adjusts the caching strategy and transmission power to the optimal balance state based on changes in correlation distance. In addition, when the correlation distance increases to a certain extent, the EE of all caching strategies will decrease. The increase in correlation distance will inevitably lead to an increase in transmission power, which will result in an increase in power consumption and interference between vehicles. Furthermore, the effectiveness and superiority of the proposed joint optimization method were verified.

## Conclusion

In this paper, we propose a joint optimization method to maximize the EE of UAV-assisted IoV. Firstly, based on the interaction between caching strategy and transmission power on the EE, we proposed a V2V communication threshold to establish a theoretical relationship between caching strategy and transmission power. And we derived expressions for network data offloading ratio and EE. Due to the coupling relationship between cache strategy and transmission power, we decouple it into two sub problems: cache strategy optimization and transmission power optimization. Then, we proposed a joint optimization algorithm to jointly solve the above two sub-problems. Finally, simulation experiments were conducted to compare TPJOM with TIOM and TUB, demonstrating the effectiveness of the proposed joint optimization method.

In addition, this paper focuses on considering a busy urban area or a multi-road intersection area where the vehicle position obeys a HPPP. If multiple lines are considered, road layout should be further considered. In this scenario, the network model should meet the Cox process or doubly stochastic Poisson point process. However, the network will become more complicated, which will be our future work. The research on cooperation caching for high-speed mobility and privacy security will also be our next focus.

## Data Availability

The datasets generated and/or analysed during the current study are not publicly available due to this manuscript does not report data generation or analysis but are available from the corresponding author on reasonable request.

## Appendix A

We take the first derivative of function $P_h$ on interval $0 \le q_f \le 1$, which can be calculated as

18 $$ \begin{aligned}   P_{h}^{\prime }  &  = \frac{{{\text{d}}P_{h} }}{{{\text{dq}}_{{\text{f}}} }} = \frac{{{\text{d}}P_{h} }}{{{\text{d}}\varphi _{{\text{f}}} }}\frac{{{\text{d}}\varphi _{f} }}{{{\text{dq}}_{{\text{f}}} }} \\     &  = 1 + \pi \lambda _{p} \left( {\frac{{1 - e^{{ - \varphi _{f} z^{2} }} }}{{\varphi _{f} }}} \right) + \pi \lambda _{p} q_{f} \varphi _{f}^{\prime } \left( {\frac{{\varphi _{f}^{2} z^{2} e^{{ - \varphi _{f} z^{2} }}  - 1 + e^{{ - \varphi _{{1,f}} z^{2} }} }}{{\varphi _{f}^{2} }}} \right). \\  \end{aligned}  $$

Furthermore, the second derivative of function on interval $0 \le q_f \le 1$ can be expressed as

19 $$ \begin{aligned}   P_{h}^{{\prime \prime }}  &  = \frac{{{\text{d}}^{2} P_{h} }}{{{\text{dq}}_{{\text{f}}}^{{\text{2}}} }} = \frac{{{\text{d}}P_{h}^{\prime } }}{{{\text{d}}\varphi _{f} }}\frac{{{\text{d}}\varphi _{f} }}{{{\text{dq}}_{{\text{f}}} }} \\     &  = 2\pi \lambda _{p} \varphi _{f}^{\prime } \left( {\frac{{\varphi _{f}^{2} z^{2} e^{{ - \varphi _{f} z^{2} }}  - 1 + e^{{ - \varphi _{f} z^{2} }} }}{{\varphi _{f}^{2} }}} \right) + \pi \lambda _{p} \varphi _{f}^{{\prime \prime }} \left( {\frac{{\varphi _{f}^{2} z^{2} e^{{ - \varphi _{f} z^{2} }}  - 1 + e^{{ - \varphi _{f} z^{2} }} }}{{\varphi _{f}^{2} }}} \right) \\     & \quad  + \pi \lambda _{p} q_{f} \varphi _{f}^{{\prime 2}} \left( {\frac{{\varphi _{f}^{2} z^{2} e^{{ - \varphi _{f} z^{2} }}  - \varphi _{f}^{4} z^{4} e^{{ - \varphi _{f} z^{2} }}  - 2\left( {\varphi _{f}^{2} z^{2} e^{{ - \varphi _{f} z^{2} }}  - 1 + e^{{ - \varphi _{{.j}} z^{2} }} } \right)}}{{\varphi _{f}^{3} }}} \right) \\     &  = \pi \lambda _{p} \left( {2\varphi _{f}^{\prime } \varphi _{f}  - 2q_{f} \varphi _{f}^{{\prime 2}}  + \varphi _{f}^{{\prime \prime }} } \right)\left( {\frac{{\varphi _{f}^{2} z^{2} e^{{ - \varphi _{f} z^{2} }}  - 1 + e^{{ - \varphi _{f} z^{2} }} }}{{\varphi _{f}^{3} }}} \right) + \pi \lambda _{p} q_{f} \varphi _{f}^{{\prime 2}} z^{2} e^{{ - \varphi _{f} z^{2} }} \left( {\frac{{1 - \varphi _{f}^{2} z^{2} }}{{\varphi _{f} }}} \right)^{{(1)}}  \\     & \quad  \le \pi \lambda _{p} \varphi ^{{\prime \prime }} \left( {\frac{{\varphi _{f}^{2} z^{2} e^{{ - \varphi _{f} z^{2} }}  + e^{{ - \varphi _{f} z^{2} }} }}{{\varphi _{f}^{3}  + 1}}} \right) + \pi \lambda _{p} q_{f} \varphi _{f}^{{\prime 2}} z^{2} e^{{ - \varphi _{f} z^{2} }} \left( {\frac{{1 - \varphi _{f}^{2} z^{2}  - e^{{\varphi _{f} z^{2} }} }}{{\varphi _{f} }}} \right) \\  \end{aligned}  $$

20 $$\begin{aligned} \begin{aligned} \varphi _f^{\prime }=\pi \lambda _p+2 \pi ^2 \lambda _r \gamma _0^{2 / \alpha } \pi \lambda _p r^2 \sum _{f=1}^F p_f e^{-\pi \lambda _p q_f r^2} \csc \left( 2 \pi \alpha ^{-1}\right) \alpha ^{-1} \ge 0. \end{aligned} \end{aligned}$$

21 $$\begin{aligned} \begin{aligned} \varphi _f^{\prime \prime }=-2 \pi ^3 \lambda _r \gamma _0^{2 / \alpha } \pi \lambda _p^2 r^4 \sum _{f=1}^F p_f e^{-\pi \lambda _p q_f r^2} \csc \left( 2 \pi \alpha ^{-1}\right) \alpha ^{-1} \le 0. \end{aligned} \end{aligned}$$

We take the first derivative of function $\varphi _f-q_f \varphi _f^{\prime }$ with respect to $q_f$, which can be expressed as $-q_f \varphi _f^{\prime \prime } \ge 0$. So we can come to conclusion $\varphi _f \ge q_f \varphi _f^{\prime }$. Based on conclusion $\varphi _f \ge q_f \varphi _f^{\prime }$, we have made an approximation to step (1) of Eq. (19). Through Eq. (21), we can determine that the first term in step (1) is a negative term. Therefore, we need to determine the positivity and negativity of the second term. Assuming the function $G\left( q_f\right) =1-\varphi _f^2 z^2-e^{\varphi _f z^2}$, we can obtain $G^{\prime }\left( q_f\right) =-2 \varphi _f \varphi _f^{\prime } z^2-\varphi _f \varphi _f^{\prime } z^2 e^{\varphi _f z^2} \le 0$ by taking the first derivative of $G\left( q_f\right)$ with respect to $q_f$. So the function $G\left( q_f\right)$ is a monotonically decreasing function on interval $0 \le q_f \le 1$. Furthermore, we can derive the conclusion of $G\left( q_f\right) \le G(0)=0$. Finally, we can conclude that the second derivative $\textrm{d}^2 P_h / \textrm{d} q_f^2 \le 0$ is on the interval $0 \le q_f \le 1$. Therefore, the objective function $P_h$ is a concave function on interval $0 \le q_f \le 1$, then the problem is a standard convex optimization problem. The proof of Proposition 1 is completed.
